# Supplementary material for: Subjective Wellbeing and Related Factors of Older Adults Nine and a Half Years after the Great East Japan Earthquake: A Cross-Sectional Study in the Coastal Area of Soma City
Source: Int J Environ Res Public Health. 2022 Feb 24;19(5):2639. doi: 10.3390/ijerph19052639 (PMC8910374; doi:10.3390/ijerph19052639)
Supplement: Supplementary file 1 [file ijerph-19-02639-s001.zip › ijerph-1599889-supplementary.pdf]

September 1st, 2020

Survey on Living Conditions and Wellbeing in Soma City Following the Great East Japan Earthquake

Professor Seiji Yasumura, Department of Public Health, Fukushima Medical University School of Medicine

Please answer the following questions by choosing the option that best applies to you, without deliberating much over your answers. Please make sure to answer as many questions as possible.

Q1 Write down your age as a number.

( ) years old as on September 1, 2020

Q2 Circle one option that describes your gender.

(1) Male (2) Female

Q3 Circle all options that best describe who you are currently living with.

(1) Living alone
(2) With a spouse (husband or wife) or partner
(3) With children (including in-laws)
(4) With parents (including in-laws)
(5) Other ( )

Q4 Circle one option that best describes your current residence.

(1) Privately owned house (general housing area)
(2) Privately owned house (condominium in public disaster housing complexes area)
(3) Rented house or apartment
(4) Idobata Nagaya row house in public disaster housing complexes
(5) Independent housing in public disaster housing complexes
(6) Other ( )

Q5 Circle one option that best describes how long you have resided in Soma City.

(1) Since birth (2) Less than one year (3) One to five years
(4) Five to ten years (5) Ten to twenty years (6) Twenty years or more

Q6 How is your current health condition? Circle one option that best applies.

(1) Very good (2) Good (3) Normal (4) Bad (5) Very bad

Q7 Circle either 1 or 2 based on the feelings that you currently have.

	Question	Yes	No
1	Do you feel that life keeps getting worse as you get older?	1	2
2	Are you as energetic as you were one year ago?	1	2
3	Do you ever feel lonely?	1	2
4	Do you find that little things bother you recently ?	1	2
5	Are you satisfied with your interactions with friends and relatives?	1	2
6	Do you feel that you have become less useful as you get older?	1	2
7	Do you ever worry so much that you cannot sleep?	1	2
8	As you age, has life become better than you thought it would be?	1	2
9	Do you ever feel that life is not worth living?	1	2
10	Are you as happy now as you were when you were younger?	1	2
11	Is there a lot to be sad about in your life?	1	2
12	Do you have many fears and worries?	1	2
13	Do you get angry more frequently than you used to?	1	2
14	Do you feel that life is hard for much of the time?	1	2
15	Are you satisfied with your current life?	1	2
16	Do you take things seriously?	1	2
17	Do you easily get upset when you encounter troubles or worries?	1	2

Q8 Are you satisfied with your dietary habits (everyday meals)? Circle one option that best applies.

(1) Very satisfied (2) Somewhat satisfied (3) Not very satisfied (4) Not satisfied

Q9 How often do you eat with friends, family, relatives, or other individuals? Circle one option that best applies.

(1) Almost every day (2) Four-five days per week (3) Two-three days per week

(4) Once per week (5) Once or twice per month (6) Rarely

Q10 Circle one option that best describes your abilities in situations in which you chew foods.

(1) Can chew any foods
(2) Cannot chew some foods
(3) Cannot chew many foods
(4) Cannot chew any foods

Q11 How many days per week do you eat the following ten food groups?

Circle one option from 1 to 4 that best describes your intake over the past week.

Question	Almost every day	Once every two days	Once or twice per week	Almost never
Seafood (Fresh fish, dried fish, kamaboko, chikuwa, hanpen, etc.)	1	2	3	4
Meat (Fresh meat, bacon, ham, sausage, etc.)	1	2	3	4
Eggs (Chicken eggs, quail eggs)	1	2	3	4
Dairy milk (Yogurt included) *Does not include milk coffee	1	2	3	4
Soybean products (Tofu, natto, fried tofu, miso, soybean flour, etc.)	1	2	3	4
Brightly colored vegetables (Carrots, spinach, tomatoes, etc.)	1	2	3	4
Seaweed (Nori, wakame, kelp, mozuku, mekabu, etc.)	1	2	3	4
Potatoes (Potatoes, sweet potatoes, taro, long potatoes, etc.)	1	2	3	4
Fruits (Fresh or canned fruit) *Does not include tomatoes	1	2	3	4
Oils/fats (Fried foods, tempura, butter, margarine, etc.)	1	2	3	4

Q12 to 14 ask about your family and relatives. Circle one option that best applies.

Q12 How many family members or relatives do you see or hear from at least once a month?	None	One	Two	Three to Four	Five to Eight	Nine or more
Q13 How many family members or relatives do you feel at ease with that you can talk about private matters?	None	One	Two	Three to Four	Five to Eight	Nine or more
Q14 How many family members or relatives do you feel close to such that you could call on them for help?	None	One	Two	Three to Four	Five to Eight	Nine or more

Q15 to 17 ask about your friends. Circle one option that best applies.

Q15<br>How many friends do you see or hear from at least once a month?	None	One	Two	Three to Four	Five to Eight	Nine or more
Q16<br>How many friends do you feel at ease with that you can talk about private matters?	None	One	Two	Three to Four	Five to Eight	Nine or more
Q17<br>How many friends do you feel close to such that you could call on them for help?	None	One	Two	Three to Four	Five to Eight	Nine or more

Q18 Do you wish to continue living in your current home region? Circle one option that best applies.

(1) Yes (2) No (3) Unsure

⇨ If you selected (1) or (2), please share your reasons in the space below.

Q19 Do you consider solitary death (passing away without anyone's care and being discovered afterward) to be a personally relevant issue? Circle one option that best applies.

(1) Very much (2) Somewhat (3) Somewhat not

(4) Not at all (5) Unsure

Q20 Circle either 1 or 2 based on your current daily living conditions.

	Question	Yes	No
1	Can you use public transportation (bus or train) by yourself?	1	2
2	Are you able to shop for daily necessities?	1	2
3	Are you able to prepare meals by yourself?	1	2
4	Are you able to pay bills?	1	2
5	Can you handle your own banking?	1	2
6	Are you able to fill out forms for your pension?	1	2
7	Do you read newspapers?	1	2
8	Do you read books or magazines?	1	2

	Question	Yes	No
9	Are you interested in news stories or programs dealing with health?	1	2
10	Do you visit the homes of friends?	1	2
11	Are you sometimes called on for advice by friends or family?	1	2
12	Are you able to visit sick friends?	1	2
13	Do you sometimes initiate conversations with young people?	1	2

Q21 Over the past month, did you get enough rest while sleeping? Circle one option that best describes your quality of sleep.

(1) Sufficient (2) Moderate

(3) Inadequate (4) None

Q22 Do you regularly visit a hospital or clinic (doctor's office, dentist's office) for illness or injury? (Includes house calls and visits.)

(1) Yes ⇨ If possible, please describe your diagnosis below.

(2) No

Q23 Circle all options that describe your experiences following the 2011 earthquake.

(1) Displacement
(2) Living separately with family
(3) Living together with family
(4) Personal health deterioration
(5) Deterioration of a family member's health
(6) Caregiving for a family member
(7) Divorce/separation/loss of spouse/partner
(8) Loss of a family member other than spouse/partner
(9) Loss of an important non-family individual
(10) Unemployment
(11) Financial hardships
(12) Difficulties in interpersonal relations
(13) Other (   )

Q24 How do you feel about your current financial lifestyle? Circle one option that best applies.

(1) Struggling (2) Somewhat struggling (3) Normal

(4) Somewhat comfortable (5) Comfortable

Q25 Which sources do you regularly rely on for information? Circle three types of media that you use most frequently, from the list below.

(1) Local newspapers (Fukushima Minpo, Fukushima Minyu, etc.)

(2) National newspapers (Yomiuri Shimbun, Asahi Shimbun, Mainichi Shimbun, etc.)

(3) NHK television

(4) Private local broadcast television (Fukushima Television, TBC, etc.)

(5) Private national broadcast television (Fuji TV, Nippon TV, TV Asahi, TBS, etc.)

(6) Radio

(7) Internet sources (including cellphones and smartphones)

(8) Magazines or books

(9) Local government publications (including prefectural bulletin)

(10) Word of mouth (from friends, acquaintances, etc.)

(11) Other ( )

Q26 Circle one option that best describes your educational attainment.

(1) Up to junior high school (2) Up to high school

(3) Junior college or vocational school (4) University or graduate school

Q27 Write down your height and weight in the spaces below in numerical format.

Height: ( ) centimeters Weight: ( ) kilograms

Please feel free to write how you feel about life in Soma City after the earthquake, as well as any feedback you may have about this survey.

The survey ends here. Thank you for your cooperation.
